# Bone-like Collagen Matrices Through Rapid Intrafibrillar Mineralisation

**DOI:** 10.3390/jfb17070344

**Published:** 2026-07-16

**Authors:** Michael Eugene Doyle, Qiancheng Zhang, Brian J. Rodriguez, Kenneth Dalgarno, Ana Marina Ferreira

**Affiliations:** 1 School of Engineering, Newcastle University, Newcastle upon Tyne NE1 7RU, UKkenny.dalgarno@newcastle.ac.uk (K.D.); 2 School of Physics and Conway Institute of Biomolecular and Biomedical Research, University College Dublin, D04 V1W8 Dublin, Ireland; qiancheng.zhang@ucd.ie (Q.Z.); brian.rodriguez@ucd.ie (B.J.R.)

**Keywords:** collagen, biomineralisation, biomimetic, bone, scaffolds

## Abstract

An innovative strategy for collagen self-assembly with accelerated intra and extrafibrillar mineralisation is introduced to generate bone scaffolds with biomimetic properties. This method, termed Rapid Fibrillogenic Mineralisation (RFM), leverages coprecipitation with 10× Simulated Body Fluid (10× SBF) during fibril formation to maximise nucleation, particularly within intrafibrillar zones at molecular termini. Densification is achieved within minutes via plastic compression driven by capillary action, producing bone-like scaffold density without compromising the collagen matrix. Transmission electron microscopy confirms intrafibrillar hydroxyapatite crystals within 15 min, while X-ray diffraction demonstrates distinct HA peaks across groups. Scanning electron microscopy verified extrafibrillar mineralisation after 4 h, with saturation by 6 h, yielding ‘nanoflower’ crystal clusters. Infrared spectra showed increased carbonate content over time, indicating lattice substitutions characteristic of natural bone. Enhanced mineralisation translated into significant mechanical gains as Dynamic Mechanical Analysis revealed compressive moduli approaching cancellous bone (up to 283 ± 31 MPa). In addition, a decrease in the piezoelectric coefficient occurs with increased mineralisation process, highlighting the effects of mineral inclusions on collagen fibre composition and anisotropy. Biologically, mineralised scaffolds supported cellular growth compared to collagen controls. RFM thus enables rapid, reproducible fabrication of biomimetic bone scaffolds that closely emulate native mineralisation patterns and mechanical behaviour. Beyond offering a practical route for scaffold production in tissue engineering, the process also provides new insights into bone physiology and in vitro modelling. By reshaping collagen into a synthetic echo of nature’s bone, RFM establishes a rapid approach for designing functional biomaterials with translational potential.

## 1. Introduction

With an ageing world population, there is a concurrent increase in the incidence of bone fractures and disorders, which require advanced treatments and a better understanding of bone conditions and diseases [1]. Indeed, studies targeting the understanding of human bone development and healing, including the underlying mechanisms behind the mineralisation of bone [2,3,4], increase the reliance mainly on animal-based studies. Therefore, in vitro bone tissue model capable of replicating the tissue environment with the capability to tune or standardise properties such as levels of mineralisation could significantly mitigate the use of animal studies and likely enable new levels of understanding of human bone physiology [5]. Bone comprises hundreds of components, but is predominantly made of type I collagen [6] and apatite mineral [7]. The intricate interplay between these elements varies across all 12 known levels of bone organisation [3], making for a highly complex, hierarchical structure. Macroscopic bone is a varied network of mineralised collagen fibres, with porosities averaging 79.3% for cancellous and 3.5% for cortical bone [8]. The Young’s modulus of each type of bone is 0.1–0.3 GPa [9] and 18–22 GPa [10], respectively. At the nanoscale, the relationship between the polymer and mineral phases is simultaneously highly organised and amorphous. Collagen molecules are highly organised with regular spacing, consistently accurate to the nanometre; the intrafibrillar spaces, which are 35 × 1.5 nm gaps [4], house hydroxyapatite (HA) crystals [11]. This HA then permeates other areas of the collagen extracellular matrix (ECM) as a gradient of phases, creating a quasi-cement [3]. While only a brief summation, some of these attributes of bone have proven irreparable without the aid of exogenous components foreign to natural bone.

In the rapidly developing field of tissue engineering, the best tissue scaffolds should closely mimic the characteristics of their natural biological counterparts. This goal has not yet been fully achieved for bone, primarily due to challenges in simultaneously replicating its matrix structure, mineral composition, and mechanical properties [12]. Bone primarily derives its mechanical strength from its mineral content, which has superior mechanical properties compared to collagen. Although some materials have made progress in certain aspects, they often fall short in other crucial qualities. Additionally, many of these processes can be toxic or destructive to collagen and may involve the use of foreign materials to artificially enhance their structures. Intrafibrillar minerals create pressure between collagen fibrils, while extrafibrillar minerals bind the collagen together [12,13]. Therefore, there is an existing need to engineer a quick and efficient mineralisation process capable of producing bone-like structures, with a mineralisation rate at intra and extrafibrillar levels that can be controlled, as well as promoting collagenous matrix organisation. [14] This gap offers a timely opportunity for advancing mineralised tissue models.

In this study, a pioneering method named “Rapid Fibrillogenic Mineralisation” is presented for faster and more efficient mineralising collagen scaffolds of bone-like density, whilst maintaining collagen matrix integrity. The level of mineral precipitation achieved with this bioinspired approach surpassed previously reported results in the literature [13,14,15] within very few hours, while avoiding the use of cytotoxic chemicals or destructive methods to crosslink the collagen matrix, which are commonly seen in state-of-the-art techniques [16] or commercial products [17]. Precipitated mineral size and distribution were confirmed via electron microscopy. The composition of these minerals was evaluated through a combination of crystallographic diffraction and infrared analysis. The impact of increased mineral content on the compressive moduli of the matrices was assessed, in addition to changes in fibrillar shear resistance. Scaffold interactions with cells were examined using viability and morphology staining, with Alkaline Phosphatase levels monitored up to 14 days.

## 2. Materials and Methods

### 2.1. Materials

All 10× SBF-RA reagents were obtained from Sigma-Aldrich (Gillingham, UK): NaCl (S9888), KCl (P3911), CaCl_2_·2H_2_O (C8106), MgCl_2_·6H_2_O (M2670), NaH_2_PO_4_·H_2_O (S9638), NaHCO_3_ (S6014), and Phenolsulfonphthalein (Phenol Red) Powder (P3532). Phosphate Buffered Saline (PBS) tablets (P4417) and NaOH solution 1N (1.09137) were also obtained from Sigma-Aldrich (Gillingham, UK). Rat Tail Collagen (Type I) was purchased from Lonza, USA-MD (016-1R24), Corning, UK (CF715/354236) and Cultrex, USA-MN (3447-020-01; 3443-100-01). All reagents were used as received without further purification.

### 2.2. Simulated Body Fluid (10× SBF) Solution Preparation

A modified version of 10× SBF-RA (as reported by [18] as Recipe A) was used for all mineralised samples. The stock solution is prepared as described by Mavis et al. [18], activated via a 10% NaHCO_3_ (*w*/*v*) solution with pH adjusted to 5.5. Phenol Red was also added to the stock solution for visual pH changes. Standard-concentration PBS (10 g/L) was used to wash samples and store collagen membranes post-densification, preventing dehydration and any associated morphological changes. PBS was used here rather than deionised water to prevent the unwanted dissolution of precipitated minerals. For isotonicity, piezoresponse force microscopy (PFM) and cell biocompatibility control samples were mixed with 10xPBS (100 g/L) in place of 10× SBF. Precipitation would occur upon cessation of stirring, necessitating that all 10xPBS be pipetted whilst stirring in an ice bath.

### 2.3. Rapid Fibrillogenic Mineralisation (RFM) Sample Preparation

**Figure 1:**
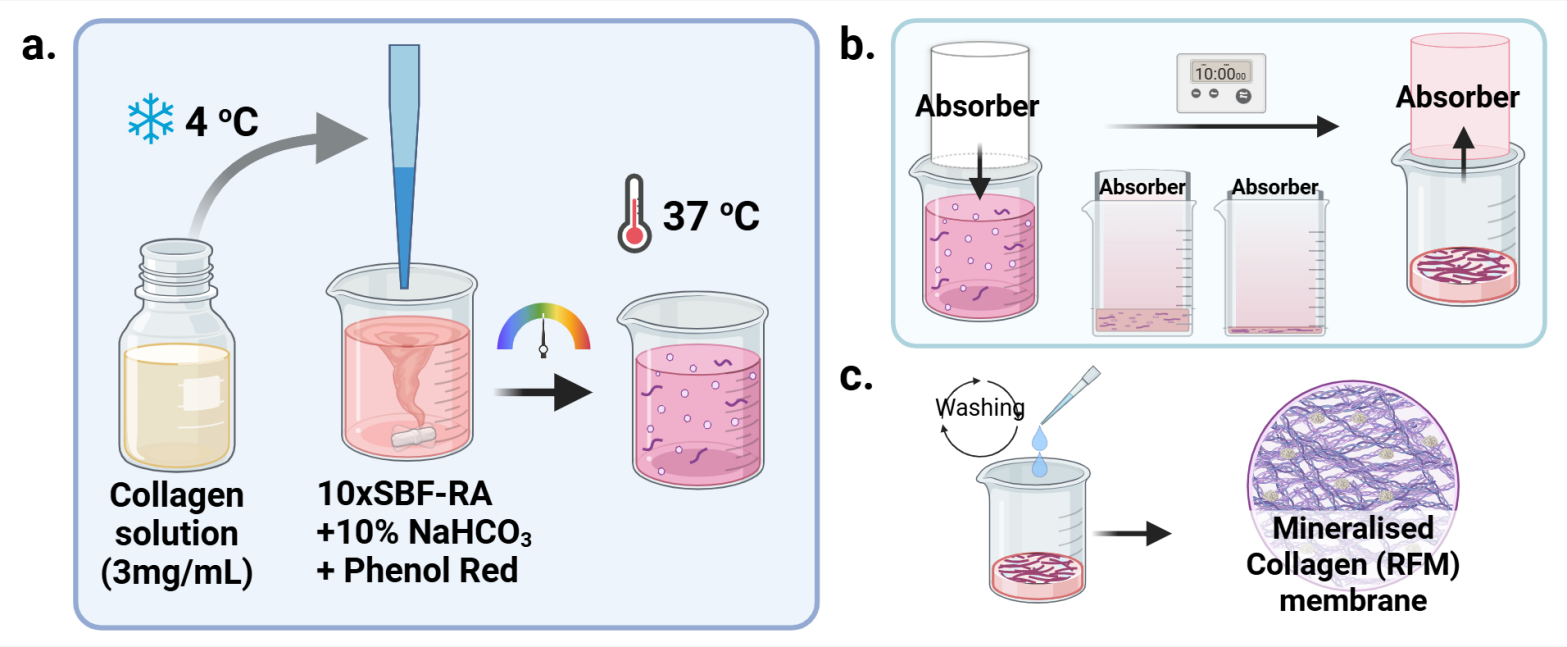
Schematic illustrations of the RFM collagen densification process: (**a**). Combining collagen and 10× SBF-RA solutions and thermal treatment. (**b**). Absorber application to promote collagen densification and fibrillar mineralisation. (**c**). Washing obtained mineralised collagen membrane (RFM, Rapid Fibrillogenic Mineralisation) with water. Created in BioRender.com/9afbpgf.

A 2:1 ratio of 3 mg/mL collagen and 10× SBF-RA stock solution was mixed, followed by the drop-wise addition of 10% (*w*/*v*) NaHCO_3_ until reaching neutral pH (also visually observed by a deep red colour) (Figure 1). From this solution, 1 mL volumes were pipetted into 24-well plates and refrigerated. When ready, the multi-wells were heated to ~37 °C on a hotplate until all samples formed set gels, approximately 10–20 min. Absorbers were then placed on top of samples, compressing them into membranes at different densification times (with 0 h corresponding to 15 min, 2 h, 4 h and 6 h). The samples were subsequently washed three times in PBS to remove the 10× SBF-RA solution from the samples. All samples were approximately 13 mm in diameter and 60 μm thick.

Collagen controls were produced using a 2:13 (*v*/*v*) ratio of 10xPBS and 5 mg/mL collagen, mixed with Phenol Red and neutralised with 0.3 M NaOH for pH adjustment. Following this, the control samples followed the same process as the mineralised samples. One control sample produced for TEM followed the above process with a few exceptions: 10xPBS was excluded as the salts present could have interfered with imaging, particularly if any were not fully dissolved or had begun to reprecipitate.

### 2.4. Preparation of Sample Absorbers

Initially, Lonza RAFT^TM^ absorbers were used for densifying collagen gels into membranes and establishing protocols. However, due to the discontinuation of Lonza RAFT^TM^ absorbers and for consistency, the protocol was adapted, and sample preparation utilised hand-crafted absorbers, prepared as described in Nature Protocols by Tysoe et al. [19]. The ‘DIY’ absorbers were supported by a grid crafted out of foam board to ensure optimal contact with the sample surfaces.

### 2.5. Physicochemical Characterisation of RFM Samples

**Chemical analysis**: A PerkinElmer Spectrum Two FTIR Spectrometer with UATR attachment and diamond ATR crystal was used to semi-quantitatively assess chemical changes related to the mineralisation of collagen membranes. Samples were passively dried in ambient conditions. The spectrum analysed was 4000–550 cm^−1^ at a resolution of 4 cm^−1^, with 16 scan accumulations, and an 8.94 mm internal aperture. The primary frequency ranges examined were those that correspond to carbonate and phosphate groups (~1500–550 cm^−1^), as calcium was not possible to detect.

**Morphological characterisation**: Morphological characterisation of extrafibrillar mineral was performed from images captured using a TESCAN VEGA3 Scanning Electron Microscope (SEM) under a high vacuum and subjected to high voltage (8 kV), at a working distance 15 mm. Samples were fixed by overnight immersion in a solution of 2% (*w*/*v*) Glutaraldehyde in 0.1 M Sorensen’s Phosphate Buffer, then washed twice for 15 min in PBS. The samples were then progressively dehydrated in ethanol dilutions of 25%, 50%, and 75% for 30 min each, followed by two 60 min washes in pure ethanol. Afterwards, samples were placed in a Bal-tec Critical Point Drier, mounted onto aluminium stubs with adhesive carbon discs, and finally sputter-coated with a 15 nm layer of gold using a Polaron SEM Coating Unit. In addition, internal slices of collagen membranes were examined using a Hitachi HT7800 Transmission Electron Microscope (TEM), paired with the EMSIS Radius software (version 2.1, EMSIS, Münster, Germany), integrated with an EMSIS CMOS Xarosa camera. Internal slices refer to ultrathin sections obtained from within the bulk of the sample, excluding surface regions, to minimise preparation artefacts and ensure representative fibrillar measurement. Initial fixation was by immersion in a solution of 2% (*w*/*v*) Glutaraldehyde in 0.1 M Sorensen’s Phosphate Buffer, then a secondary fixation in 1% (*w*/*v*) Osmium Tetroxide solution. Dehydration was achieved with 25%, 50%, and 75% Acetone for 30 min each, followed by two 60 min treatments in 100% Acetone. Next, the samples were gradually impregnated with 25%, 50%, and 75% Resin in Acetone for 60 min each, then 100% resin over 24 h (changed three times), before finally being embedded in 100% Resin at 60 °C for 24 h. Sections 0.5 μm thick were then cut from the embedded sample and stained in 1% (*w*/*v*) Toluidine Blue in 1% (*w*/*v*) Borax solution. A diamond knife on a Leica EM UC7 was used to cut these sections down to approximately 70 nm in thickness before being stretched with Chloroform and mounted on Pioloform-filmed copper grids.

**Atomic force microscopy (AFM) and lateral piezoresponse force microscopy (LPFM)**: measurements were conducted to assess the surface morphology and nanoscale electromechanical response of mineralised (RFM 6 h) and control collagen films, respectively. All measurements were performed on an Asylum Research MFP-3D AFM using Pt-coated Asyelec-01-R2 AFM probes. Samples were dried in a fume cupboard under negative air pressure and mounted on glass slides before measurement. Randomly selected 50 × 50 µm areas from a 6 h RFM sample and a collagen control were scanned at a resolution of 256 × 256. Multiple (*n* > 10) 5 × 5 µm sub-regions were analysed for each of these two groups with an AC voltage (Vac) of 60 V at 30 kHz. To determine the effective shear piezoelectric coefficient (d_15_), Vac sweep measurements were performed on fibrils that were oriented orthogonal to the cantilever axis and exhibited high LPFM amplitude response. At each selected location, a series of Vac = 0–60 V sweeps (>40 locations) were applied, and the linear slope of the amplitude–voltage relationship was taken as the local d_15_. The lateral inverse optical lever sensitivity (invOLS) was calculated from the deflection invOLS multiplied by 3H/2L, where L is the cantilever length, and H is the tip height, following the geometric conversion method for torsional deflection calibration [20]. The AFM probe maintained its sensitivity throughout the measurements, which was verified by re-measuring the first sample at the end of the sequence. The mean LPFM amplitude increased by approximately 9%, indicating no degradation in probe performance.

**Assessment of nucleated (HA) mineral crystallinity**: X-Ray Diffraction (XRD) analysis was performed to assess the crystallinity of HA mineralised on, or within, dense collagen scaffolds, allowing evaluation of the induction time of intrafibrillar minerals. The following are the specifications used for analysis: Diffractometer used: XRD2—PANalytical X’Pert with Xcelerator detector. Experimental conditions: Samples were supplied as powders of flakes, which were deposited directly onto zero background holders for measurement. Incident optics: Soller slit = 0.04 rad, Programmable Divergence Slit, fixed to 6 mm, Beam mask = 20 mm, and Anti-scatter slit = 2°. Diffracted optics: Soller slit = 0.04 rad, Ni filter. 2ϴ range: 20–100°, step size = 0.0334°, and 1.5 s step-1. It should also be noted that carbon content was also excluded from XRD analysis. Samples were supplied as films, which had been dried in a fume cupboard with negative air pressure onto glass slides.

**Mechanical studies of RFM samples via DMA**: A Perkin Elmer Dynamic Mechanical Analyzer (DMA) 8000 was used to assess the compressive moduli of dry mineralised collagen samples through sinusoidal deformation. The experiment type was a temperature/time scan with a single strain and 1 Hz frequency, ranging from ambient temperature (22–26 °C) up to 180 °C over a <80 min period. Values for compressive moduli were taken as the value presented at physiological temperature (37 °C) and were calculated in software based on the sample geometry and stiffness. For mechanical analysis, the samples were also dried in a fume hood under negative air pressure. Samples were shaped into rectangular cuboid shapes (approximately 2.5 mm × 1.5 mm × 1.0 mm) using a digital micrometre for consistency.

### 2.6. Cell Culture and Cytocompatibility Analysis

TERT human mesenchymal stem cells (hMSCs-Y201 [15], kindly provided by York University) were cultured in basal media DMEM (high glucose without glutamine), 10% heat-inactivated foetal bovine serum, 2 mM L-Glutamine and 1% penicillin and streptomycin. A cell density of one million cells was cultured in T-75 tissue culture flasks (passage 78), regularly passaged every 3 days by using trypsin at a ratio of 1:3 once a confluence of 70–80% was achieved. Cells were incubated in 5% CO_2_ humidified atmosphere at 37 °C.

**Assessment of cell viability and metabolic activity**: The assays were performed following standard protocols and manufacturer guidelines. PrestoBlue^®^ assay (ThermoFisher Scientific, Waltham, MA, USA) was used to evaluate the metabolic activity and viability following 1, 7, and 14 days of culture. For this, a PrestoBlue working solution was prepared by mixing PrestoBlue^®^ reagent in cell culture media in a ratio of 1:9, as per manufacturer instructions. The cell culture media was removed and substituted with 1 mL of PrestoBlue^®^ working solution, then the samples were incubated for 60 min at 37 °C in a 5% CO_2_ atmosphere protected from light. Following the incubation time, 100 μL of the PrestoBlue solution was transferred from each sample’s well to a clear 96-well plate, and fluorescence readings were taken (excitation 544 nm, emission 590 nm) using a FLUOstar^®^ Omega (415-0567) microplate reader (BMG Labtech, Ortenberg, Germany). Measurements were made in triplicate.

Cell viability was assessed via Live/Dead^®^ Viability/Cytotoxicity Kit (ThermoFisher Scientific) at different time points, following manufacturer instructions. For this, 2 μM calcein acetoxymethyl and 4 μM EthD-1 were prepared in PBS from stock solutions provided by the manufacturer and protected from light with aluminium foil. Cell media was removed, and substrates were washed with DPBS. Then Live/Dead^®^ working solution was added, and the samples were incubated for 30 min at room temperature and protected from light. Following incubation, the working solution was replaced with DPBS and visualised under EVOS™ Microscope M5000 Imaging System (Invitrogen™, Carlsbad, CA, USA) at 10× magnification (calcein fluorescence as green and EthD-1 in red).

**Cytoskeleton staining to assess cell morphology**: Cell-seeded constructs were fixed at different time points with 4% paraformaldehyde solution (preheated at 37 °C) for 10 min, followed by three washing steps with DPBS. Samples were then incubated with Phalloidin-tetramethylrhodamine B isothiocyanate peptide (ThermoFisher) in 0.1% Tween 20/DPBS (1:500 dilution) for 30 min at room temperature and then washed with DPBS. One drop of DAPI mounting (4,6-diamidino-2-phenylindole, Sigma-Aldrich) was added to the samples and imaged with EVOS™ Microscope M5000 Imaging System (Invitrogen™) at 10× magnification (nucleus DAPI fluorescence as blue, and cytoskeleton in red).

**Assessment of Alkaline Phosphatase Activity**: Alkaline Phosphatase (ALP) activity was assessed using the p-nitrophenol assay. Nitrophenyl phosphate (pNPP; Sigma), which develops to a yellow end-product, p-nitrophenol, when hydrolysed by alkaline phosphatase. Samples’ culture media were removed and then washed three times with DPBS. Samples were fixed with 4% of Paraformaldehyde (Sigma Aldrich, UK) fixative solution (preheated at 37 °C) and left for 10 min. Then, the fixative solution was removed, samples were washed twice with DPBS and the alkalinised with 0.1 M Tris, pH 8.3. Samples were incubated with 500 µL of Alkaline Phosphatase Yellow (pNPP, Sigma-Aldrich) liquid substrate system at 37 °C for 45 min until a yellow colour developed. Then, 100 µL of each sample solution was transferred into a 96-well plate in triplicate. The ALP measurements were recorded in triplicate with a FLUOstar^®^ Omega microplate reader (BMG Labtech, Germany) at 405 nm. A standard curve for absorbance was prepared by serial dilutions of 50 µmol/L p-NP p-nytrophenol (Sigma, UK) solution in 0.1 M Tris (pH 8.3) covering the range 0–200 nmolpNP/mL.

### 2.7. Statistical Analysis

Tests were performed on triplicates of each sample, and results were presented as mean value ± standard deviation or standard error. Two-way ANOVA with Bonferroni post-hoc test was performed with GraphPad Prism™ 11 software (GraphPad software, La Jolla, CA, USA) using a level of significance of *p* < 0.1 (*), *p* < 0.05 (**), *p* < 0.001 (***) and *p* < 0.0001 (****).

### 2.8. Use of Generative Artificial Intelligence (GenAI)

**Figure 2:**
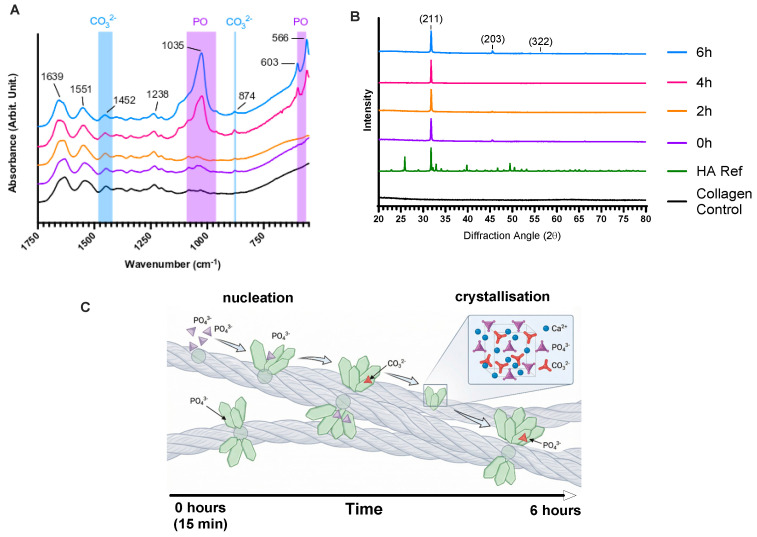
Spectra of RFM samples vs. collagen controls. (**A**). FTIR with samples separated to highlight those with significant differences. Purple regions correspond to HA-PO (Phosphate) groups, and blue regions relate to carbonate. (**B**). XRD readings of RFM samples with HA crystal reference spectrum. Peaks are labelled with their corresponding crystal lattices. The same colour code has been used for FTIR-ATR spectra and XRD for collagen control (black), purple (0 h), yellow (2 h), pink (4 h) and blue (6 h). (**C**). Schematic representation of HA crystal nucleation along exposed collagen fibrils, incorporating phosphates (PO_4_^3−^) and carbonate groups (CO_3_^2−^) into the crystal lattice (Created by FigureLabs.ai/FL-PUB-20260621-73G2G3).

**Figure 8:**
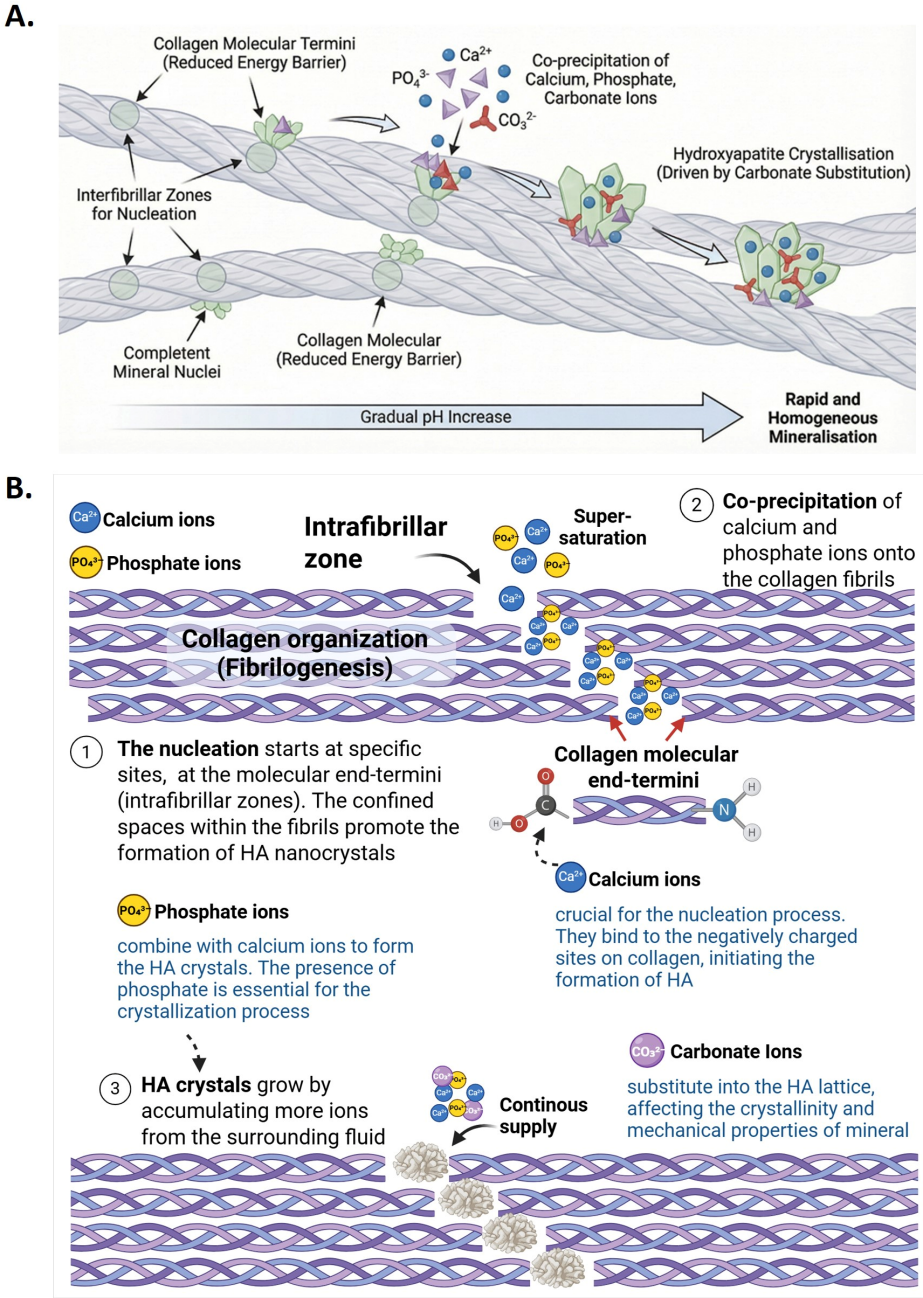
A schematic illustration demonstrates: (**A**). The rapid mineralisation of collagen fibres, showing nucleation at interfibrillar zones, co-precipitation of calcium and phosphate ions, and hydroxyapatite crystallisation enhanced by carbonate ion substitution, with collagen termini promoting efficient mineral formation (Created by FigureLabs.ai/FL-PUB-20260621-YXQ3BW). (**B**). Biomineralisation process, step-by-step, initiated by the nucleation (1) at the interfibrillar zones, (2) the co-precipitation of calcium and phosphate ions and (3) the hydroxyapatite crystallisation driven by the carbonate ion substitution. Created in BioRender.com/9afbpgf.

The authors declare the use of FigureLabs.ai and BioRender.com to create the illustrations (e.g., Figures 2 and 8).

## 3. Results

### 3.1. Chemical Characterisation via FTIR and XRD

Beyond 2 h, a notable increase is seen in the HA-PO (Phosphate) regions highlighted in purple, and a minor increase in the carbonate regions highlighted in blue (Figure 2A). Of note is the 2 h RFM sample (yellow-line); this spectrum deviates from the trend established in all other samples from this group in that it sees a comparative decline in absorbance from around 1500 cm^−1^, though this is likely sample variance rather than a considerable difference. This XRD plot (Figure 2B) compares multiple RFM samples against a reference hydroxyapatite pattern (green) and a collagen control (black). The collagen control sample expectedly produced a reading with no apparent apatite peaks, only a broad, shallow hump at the lower diffraction angles; this is typical for collagen gels. The green trace represents standard crystalline HA, showing multiple sharp peaks across the spectrum, including the characteristic apatite reflections, used to benchmark phase identification. All mineralised RFM showed the characteristic HA peak near ~31–32° (211), confirming the apatite formation at the different densification times. At early stages of the RFM process (a few minutes up to two hours, purple and orange), early-stage mineralisation or smaller crystallite size is detected as per the presence of the HA peak (211, 2θ~31.7°) with limited secondary reflections. By increasing the densification time up to 6 h, the increasing peak sharpness (211) and the appearance of higher-angle reflections (e.g., 203, 2θ~48–49°|322, 2θ~55–56°) indicate progressive crystallinity and mineral maturation. In biomimetic mineralisation systems, observing these characteristic peaks together indicates that the mineral phase is not merely amorphous calcium phosphate but has evolved into structurally ordered hydroxyapatite. Compared with the reference (green), the mineral phases appear bone-like and nanocrystalline, consistent with biomimetic mineralisation.

### 3.2. Mechanical Studies of RFM Samples via DMA

**Figure 3:**
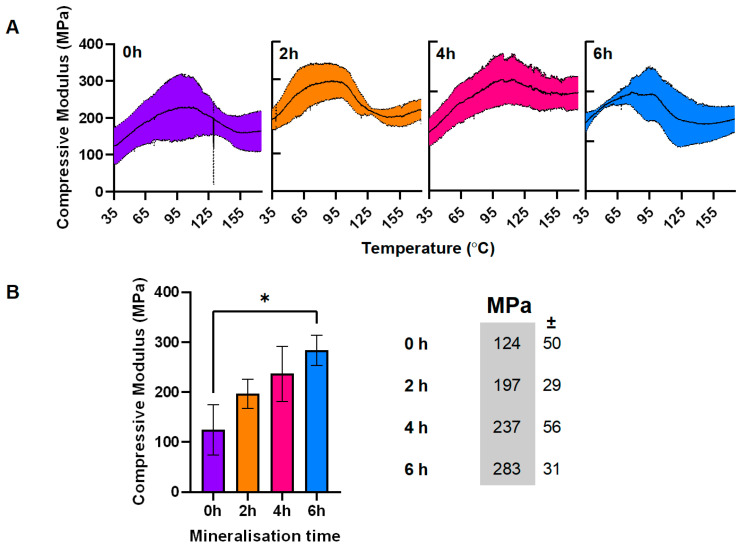
Compressive DMA output data. (**A**). Plots from 35–175 °C, collected at <1 °C intervals; black line indicates the mean while colour area indicates the associated error (Standard deviation). (**B**). Mean and standard deviation of the compressive moduli for each RFM sample group taken at 37 °C, with corresponding data tabulated on the right-hand side. Two-way ANOVA using the Bonferroni method was used for sample comparison. A significant difference was found only between the 0 h and 6 h samples (* *p* = 0.0388). (*n* = 3: per group).

All sample types had moduli within the range of cancellous bone, 120–1700 MPa [21], increasing linearly with mineralisation time when taking the moduli at 37 °C. Approximately a 2.28× increase in mean compressive modulus was seen between the 0 h and 6 h samples (Figure 3B), marking the only statistically significant difference across all groups. Before analysis, the samples were air-dried, removing bulk water from the matrices but allowing bound and structural water to remain; these aspects can be inferred through the output data. Except for the 6 h samples, there was a gradual increase in compressive modulus up to around 100 °C for all groups (Figure 3A). Around this temperature, the remaining water will have started to evaporate. Beyond this point, there was a decline in modulus roughly equivalent to the rate of increase prior. This continued for a further 30–40 °C, after which the moduli tended to stay constant; presumably, at this stage, the matrices were fully compressed. The 4 h sample set displayed the highest moduli from 80 °C onward, despite initially performing intermediately to the 2 h and 6 h groups.

### 3.3. Morphological Studies of Collagen Biomineralisation and HA Nucleation via TEM

**Figure 4:**
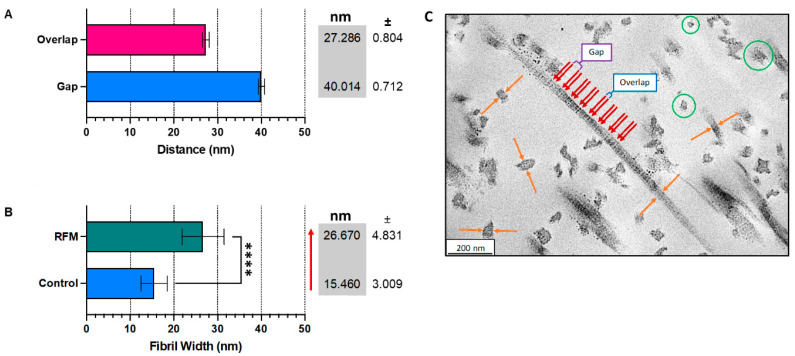
Measurements and statistical analysis of TEM images: (**A**). Intrafibrillar mineral spacing from all RFM sample groups. No significant differences were found between the gap and overlap regions within each group. (*n* = 163: Overlap; *n* = 150: Gap). (**B**) Mean fibril width measurements from TEM images with standard deviations for control and 0h-RFM. Two-way ANOVA using the Bonferroni method was used for comparison (**** *p* < 0.0001). (*n* = 921: RFM; *n* = 702: Control). (**C**) Annotated TEM image of 2h-RFM sample. Red arrows show intrafibrillar HA minerals; orange arrows show fibril edges (diameter); and green circles highlight out-of-plane fibrils.

Slices of each sample type, including a collagen control, were scanned using TEM (Supplementary Figure S1A–F) and compared for fibrillar morphology and mineral spacing. Measurements of mineral distribution and intrafibrillar spacing are consistent with the gap and overlap regions across all relevant samples (Figure 4A and Figure S1G), in agreement with where HA mineral resides in bone [22]. The mean intrafibrillar mineral spacing and fibrillar diameters between control and 0 h-RFM (thus, 15 min into the densification process) are shown in Figure 4B. These values also varied from roughly 15–30 nm between the control and the rest of the mineralised samples (Supplementary Figure S1H). While significant differences are observed between the control (non-densified) and RFM samples, no statistically significant differences exist among the RFM groups (0 h, 2 h, 4 h, 6 h). Therefore, although the collagen control was found to have significantly narrower fibrils, this was most likely due to variations in production parameters, as it is established that collagen fibrils can typically measure between 20 and 500 nm in thickness [23]. Across mineralised samples (Figure 4C), distinct black ‘dots’ can be observed along and adjacent to the fibril lengths. In-plane fibrils are the long shapes running across the images; the out-of-plane fibrils are circular in shape (perpendicular view), or short in length with an irregular shape. The in-plane fibrils often appear to fade out of view before terminating; this is the fibril exiting the plane of view, and as such, the length of fibrils is potentially much greater than can be determined using TEM measurements (the sample slice viewed is approximately 70 nm thick). Fibril lengths were not considered.

### 3.4. Morphological Studies of Collagen Biomineralisation via SEM

**Figure 5:**
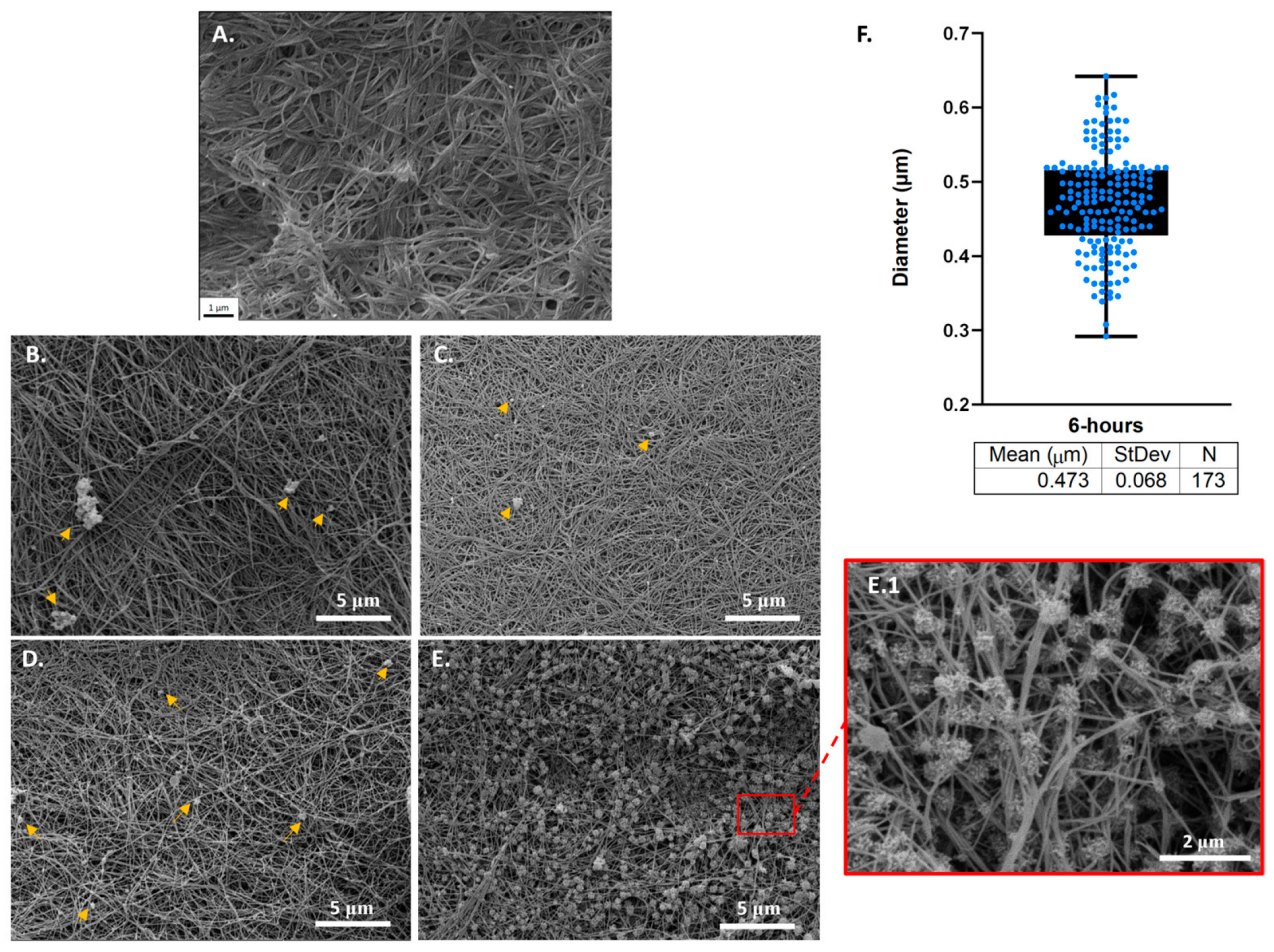
SEM images: (**A**) collagen control and RFM samples; (**B**) 0 h; (**C**) 2 h; (**D**) 4 h (yellow arrows highlight precipitated mineral); (**E**) 6 h; with (**E1**) inset image showing fibrillar mineralisation and distribution at higher magnification, and (**F**) graph of 6h-RFM particle diameters (*n* = 173; mean ± St.Dev.).

SEM imaging of RFM sample surfaces shows a direct correlation between mineral development and mineralisation time (Figure 5). The shortest mineralisation (Figure 5B, 0 h) resembles the control sample (Figure 5A). At 2 h (Figure 5C), there is the suggestion of perceptible precipitation sparsely distributed across the surface (indicated by the yellow arrows). By the fourth hour (Figure 5D), minerals are visible and distributed more frequently than in the prior sample. At hour 6 (Figure 5E1), widespread mineralisation is observed throughout the image; it is also clear that nucleation occurred homogeneously throughout the sample as it extends beneath the top surface (Supplementary Figure S2A–F). Nucleation sites within these samples appear to be along and between collagen fibrils. On close inspection, the precipitates are revealed to be ‘nano-flowers’ as described by Munyemana et al. [24] (Figure 5E1), measuring 0.473 μm ± 0.068 μm on average across 173 separate precipitates.

### 3.5. Morphological and Piezoelectric Studies of Collagen Biomineralisation via PFM

**Figure 6:**
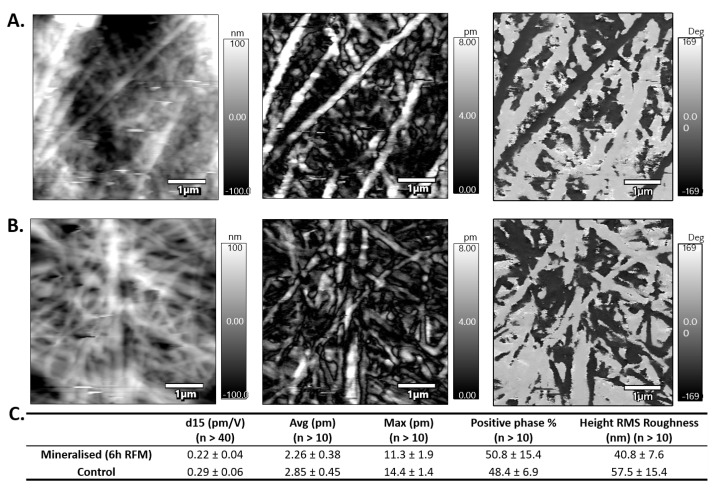
Topography, LPFM amplitude, and LPFM phase image of (**A**) collagen control and (**B**) 6 h RFM sample. (**C**) Statistical comparisons between the 6 h RFM sample and collagen control as determined by AFM and LPFM analysis.

The nanoscale electromechanical behaviour of both control and RFM (6 h) collagen was assessed by AFM and LPFM. The topography images (Figure 6A,B) reveal well-defined fibrillar structures in both samples, consistent with those observed by SEM. Quantitative analysis (*n* = 10) of the surface roughness (Figure 6C), calculated from localised nanoscale regions, indicates that the control collagen surface exhibits greater variability and tends to be slightly rougher (57 ± 15 nm) than the RFM sample (41 ± 8 nm). Mineralisation introduces both intra and interfibrillar mineral, which can locally reduce nanoscale height variations by filling gap regions within fibrils and spaces between fibrils. While larger extrafibrillar clusters may increase roughness at higher length scales. This scale-dependent behaviour is consistent with the hierarchical structure of mineralised collagen [25].

The corresponding LPFM amplitude images exhibit strong piezoresponse signals aligned along the fibril length, confirming that the electromechanical activity follows the collagen fibre orientation seen in the topography. More than ten 5 × 5 µm scans were collected for both control and 6 h RFM. The control sample shows an average amplitude of 2.85 ± 0.45 pm and an average maximum amplitude of 14.4 ± 1.4 pm, whereas the RFM sample shows an average amplitude of 2.26 ± 0.38 pm and an average maximum amplitude of 11.3 ± 1.9 pm (Figure 6C), revealing a reduction in overall piezoresponse after mineralisation. In addition to mapping the amplitude response, point Vac sweep measurements were performed to quantitatively determine the effective shear piezoelectric coefficient (d_15_). After LPFM imaging, the tip was positioned on high-response fibrils oriented orthogonally to the cantilever axis, and Vac sweeps from 0 to 60 V were applied. The fitted slope yields a d_15_ of 0.29 ± 0.06 pm V^−1^ for the control sample and 0.22 ± 0.04 pm V^−1^ for the RFM sample, consistent with the LPFM amplitude data showing a lower piezoresponse after mineralisation.

The LPFM phase images indicate the local polarisation direction of fibrils. For natural collagen bundles, approximately half of the fibrils are polarised “up” towards the top of the image and the other half “down,” yielding an overall nonpolar macroscopic structure while retaining strong local piezoelectricity. This characteristic reflects the inherent polarity alternation within native collagen assembly [26]. Both control and RFM samples exhibit this expected behaviour, with positive phase percentages of 48.4 ± 6.9% and 50.8 ± 15.4%, respectively, confirming similar fibril orientation distributions.

### 3.6. Cytocompatibility Studies of RFM Substrates

**Figure 7:**
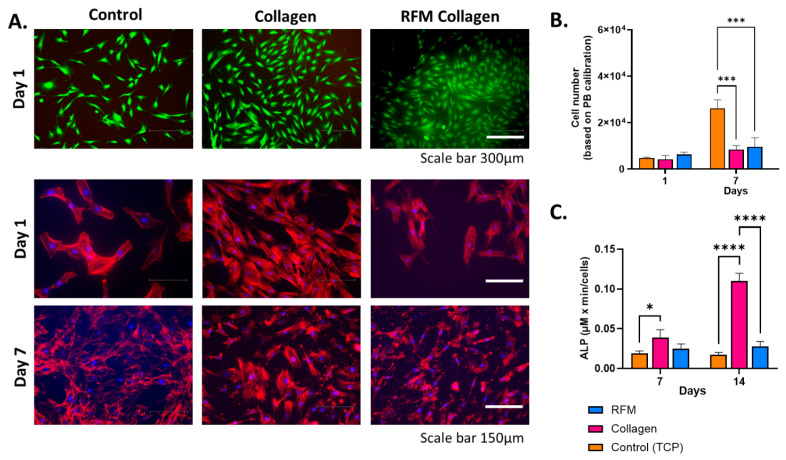
(**A**) Live–dead images (**top** images; green: live cells; red: dead cells) at day 1 and cell morphology (**bottom** images; blue: DAPI nucleus staining; red: cytoskeleton/actin filaments, phalloidin staining) of cells seeded on tissue culture plastic, collagen controls, and 6 h RFM collagen samples at day 1 and 7 days. (**B**) Cell proliferation over time at 1 and 7 days for each group (mean ± St.Dev). (**C**) ALP expression for each sample group normalised by the number of cells at 7 and 14 days. Two-way ANOVA with Bonferroni correction were used to compare the data in both cases. (* *p* = 0.0102, *** *p* < 0.001, **** *p* < 0.0001) (*n* = 3).

Live–dead staining confirmed that none of the samples analysed was cytotoxic to cells 24 h after seeding (Figure 7A). Morphologically, all cells had a spindle-like shape within the first 24 h; however, by day 7, slight differences arose, and those in collagen-based samples changed to have a more ‘star-like’ appearance. Cell proliferation was assessed at 1 and 7 days (Figure 7B). On day 1, no differences in calculated cell number (based on metabolic activity calibration) were found between controls and RFM-6h samples. By day 7, the tissue culture plastic (TCP) control had significantly increased cell growth compared to the collagen control and 6 h RFM samples. Alkaline Phosphatase (ALP), a cellular marker associated with increased osteoblast activity and biomineralisation, showed over a two-fold increase when cells were grown in collagen substrates (Figure 7C) by day 14, while ALP levels were maintained relatively low for both the TCP control and the 6 h RFM sample.

## 4. Discussion

Extrafibrillar mineral formation was evident by SEM, with precipitates appearing as early as 4 h and evolving by 6 h into ~500 nm ‘nanoflower’ clusters characteristic of biomimetic HA [24]. This late-stage growth coincided with a marked increase in the carbonate and HA phosphate PO-groups detected by FTIR (Figure 2A), indicating enhanced mineral content. Complementary TEM imaging confirmed the presence of intrafibrillar HA across all RFM groups, observed as evenly spaced dark dots along the length in-plane and aligned with the characteristic ‘gap’ and ‘overlap’ regions of collagen fibrils (Figure 4A,C). Notably, intrafibrillar crystallites were detected even in 0 h samples, which were allowed 15 min to mineralise while being compressed—underscoring the rapidity of nucleation during compression. These findings highlight the accelerated kinetics of mineral deposition achieved by dispersing collagen molecules within the mineralising solution densification process, which enables colloidal precipitates to attach uniformly along fibrils before assembling into an intact matrix [27]. XRD further validated the presence of highly crystalline HA within 15 min (0 h), with distinct sharp peaks despite partial suppression by the collagen substrate hump (2θ ≈ 20–30°; Figure 2B). The distinct intense peaks likely reflect (1) crystal orientation dictated by collagen-guided nucleation, and (2) altered crystal morphology under the highly supersaturated 10× SBF-RA environment, which accelerates growth and favours dominant crystal faces [28]. The consistently sharp and narrow peaks qualify these minerals as highly crystalline for all samples, regardless of precipitate size or quantity, indicating a consistent composition throughout crystal clusters as they develop.

Therefore, these results confirm that RFM produces consistently crystalline minerals, with fibrils acting as favourable nucleation sites [29] and carbonate substitution facilitating crystallisation (Figure 8A). As illustrated, the overall process initiates with nucleation at interfibrillar zones, followed by the co-precipitation of calcium and phosphate ions, and culminates in hydroxyapatite crystallisation facilitated by carbonate substitution. The gradual pH increase of 10× SBF-RA once activated [19] may promote a more conducive HA mineral development [30,31]. A step-by-step illustration of the intrafibrillar mineralisation process is shown (Figure 8B), proposed based on the chemical and morphological findings in this work, supported by recent literature [32,33,34]. Collagen fibrillogenesis establishes confined intrafibrillar spaces and charged molecular termini that function as preferential nucleation sites by reducing the energy required for the process to occur [35]. Under supersaturated conditions, Ca^2+^ binds to negatively charged collagen domains while phosphate ions co-precipitate along and within the fibrils (Step 2), initiating hydroxyapatite (HA) formation. TEM imaging confirmed intrafibrillar HA across all RFM groups (Supplementary Figure S1), appearing as evenly spaced dark deposits aligned with the characteristic gap and overlap regions of collagen fibrils; notably, these crystallites were already present in 0 h samples mineralised for only 15 min during compression, demonstrating rapid nucleation. Concurrently, SEM revealed extrafibrillar mineral growth (Figure 5), with precipitates visible by 4 h and evolving into ~500 nm nanoflower clusters by 6 h, indicating progressive mineral accumulation. HA crystals then grow through continuous ion supply and carbonate substitution within the lattice (Step 3), enhancing biomimetic composition and influencing mechanical behaviour. FTIR analysis showed a marked increase in carbonate and phosphate bands consistent with increasing mineral content (Figure 2A), while XRD (Figure 2B) confirmed highly crystalline HA within 15 min, evidenced by sharp diffraction peaks despite partial attenuation from the collagen substrate hump (2θ ≈ 20–30°). This synergy between collagen termini, pH-driven ion availability, and supersaturation underpins the remarkable speed and homogeneity of mineralisation observed, demonstrating that rapid intra and extrafibrillar mineralisation is driven by collagen-guided nucleation and carbonate-mediated crystallisation.

In addition, a direct correlation between mineralisation time and mean compressive moduli at 37 °C (Figure 3) is observed, potentially favoured by a hydraulic stiffening effect that intrafibrillar HA mineral has on collagen [36]. Before testing, the samples had been allowed to air-dry, releasing matrix-bound water, but allowing water trapped between collagen molecules to remain. Interestingly, after the temperature increased over 100 °C, the modulus trended downward (Figure 3A). This phenomenon can be attributed to collagen denaturation and the removal of the intrinsic water present in collagen molecules, which disrupts the interactions of intrafibrillar mineral. As the mineral phase is embedded within the collagen matrix, the damage to the collagen network leads to a release of residual compressive stresses on the mineral [37,38]. Damage to the collagen network, which forms the structural backbone, can weaken the overall composite material and the material’s ability to resist deformation and maintain its strength [39]. In addition, the mechanical performance of mineralised collagen reported in literature varies significantly depending on variables such as matrix and mineral density, scaffold hydration, sample dimensions, and the type and frequency of mechanical tests performed. For example, measurements have been obtained from relatively similar sample types, which range from 6.23 Pa [40] to around 60 kPa [41].

Comparing 6 h RFM samples with collagen controls using PFM revealed that mineralised samples were smoother. Surface roughness was reduced by nearly 30% from an average range of 57.49 nm to 40.81 nm (Figure 6B,C), which is attributed primarily to the distribution of large extrafibrillar mineral clusters, both intra and interfibrillar (Figure 4 and Figure 5). A similar change was found in the shear piezoelectric coefficient (d15) [42], which increases with the sample’s electrical response. This coefficient decreased from 0.29 pm/V to 0.22 pm/V with the increasing mineralisation process (6 h). This behaviour change can be attributed to the changes in the material’s composition, which affect the anisotropic properties as well as the surface charge generation and distributions [43]. This phenomenon has been reported in native tissues. For instance, Kwon et al. [44], have studied differences between the piezoresponse of wild-type (WT) and osteogenesis imperfecta (OI) mineralised collagen samples. The study found that the piezoelectric properties of WT collagen displayed a periodic profile along the collagen fibril’s structure. The piezoelectric values were higher in the gap regions, measuring 0.31  ±  0.02 pm/V, compared to the overlap regions, which measured 0.20  ±  0.01 pm/V. In contrast, the OI collagen fibrils showed statistically similar piezoelectric values in both the gap regions (0.37  ±  0.03 pm/V) and the overlap regions (0.32  ±  0.03 pm/V), despite having the same structural pattern. As described by Kwon et al., collagen piezoelectricity becomes ineffective as the collagen matrix builds a large amount of mineral on its surface, as it would play an important role at the intra or interfibrillar level in the initial primary mineralisation rather than secondary mineralisation. This phenomenon is particularly observed by the authors in tissues from OI, known as brittle disease, which presented loss of piezoelectric heterogeneity, which finally results in a failure under high mechanical strains. Considering the homogeneity observed in the RFM samples compared to the collagen samples, we see that peak deformation decreases from 1.73 nm to 1.41 nm after mineralisation. This reduction is attributed to minerals filling the intermolecular gaps between collagen fibrils, which helps strengthen the matrix, making it more resistant to shear stress [45]. Matrix organisation was unaffected by mineralisation as both sample groups delivered roughly a 50% positive phase, which relates to the relative orientation of fibres on the sample surface.

Regarding the impact of the RFM mineralisation process on human stromal mesenchymal stem cells (TERT-hMSCs, Y201), 6 h RFM substrates were non-cytotoxic and maintained cell proliferation comparable to collagen control matrices, although values remained below those detected for TCP at day 7 (Figure 7A,B). While ALP expression (a direct indicator of cellular mineralisation) in the 6 h RFM was comparable to that obtained for TCP substrates, it was significantly lower than in collagen substrates (Figure 7C), suggesting a differential regulation of proliferation and early osteogenic differentiation that warrants deeper investigation. This behaviour may be associated with the combined influence of matrix stiffness, fibrillar organisation and piezoelectric properties, which in collagen matrix appear to provide a more favourable osteoinductive environment. Future work may explore the use of primary stem cells, extended differentiation markers, and systematic variation of matrix mineralisation and mechanical properties to further understand their role in cell behaviour. As shown, the increase in the intrafibrillar HA leads to an increase in the mechanical stiffness of fibrils within the matrix [36,46], which has a direct impact on cellular signalling and behaviour [47,48,49]. The increased matrix stiffness could also have influenced the mechanotransduction and differentiation pathway of the cells [50]. In addition, recent findings by Zhang et al., show that extracellular calcium levels critically regulate how osteoblasts and stem cells coordinate collagen deposition and mineral handling [51]. Rather than independently boosting Ca^2+^ uptake or collagen expression, biomaterials should present a balanced calcium–collagen ratio that supports controlled mineralisation and appropriate cellular responses. This alignment ensures that engineered matrices more closely reproduce the conditions required for effective bone formation. Therefore, the RFM process can be advantageous when developing in vitro models to study bone physiological and pathological processes in vitro. Indeed, the matrix mineralisation levels and fibre organisation influence the mechanical and piezoelectric properties [44] and consequently the cellular response, particularly as the generation of bioelectric potentials can affect the mechanosensitive channels [52].

By generating minerals with highly biomimetic deposition patterns and physicochemical properties, RFM establishes a synthetic route to mineralised collagen that mirrors key features of biological apatite, including carbonate incorporation and crystallinity. At this early stage, RFM scaffolds already demonstrate bone-like density, mechanical competence, and cytocompatibility, underscoring their promise as synthetic bone substitutes and bone void fillers for critical-size defects. Beyond translational applications, the methodology also provides a platform for probing the fundamental mechanisms of collagen–mineral interactions under controlled conditions. Refinement of RFM, coupled with advanced fabrication, could enable realistic biomimetic bone models. Custom bioinks for 3D printing may replicate bone macrostructure, reinforced through mineral/peptide saturation to emulate cancellous bone and coated with dense layers to mimic cortical bone. Optimising parameters such as mineralisation time, ion ratios, and additive incorporation would further enhance apatite density and chemistry. For instance, 3D printing offers spatial control over vascular channels and density gradients, while controlled dehydration of hydrogel-based bioinks could boost resolution, enabling fine sub-microscale features. As macroporosity may reduce mechanical strength, composite reinforcement strategies will be essential to balance biological functionality with load-bearing performance.

## 5. Conclusions

This study establishes Rapid Fibrillogenic Mineralisation (RFM) as a quick approach for synthesising dense, mineralised collagen structure interfaces with bone-like properties in minutes to a few hours. By coprecipitating soluble collagen with 10× SBF during self-assembly and applying plastic compression, RFM uniquely achieves rapid, homogeneous mineralisation while preserving matrix integrity. Highly crystalline hydroxyapatite formed within 15 min, as evidenced by XRD analysis, with carbonate substitutions detected by FTIR and visible as extrafibrillar deposits under SEM after 4 h. The incorporation of carbonate, a hallmark of biological apatite, enhances mineral solubility and mirrors in vivo remodelling potential. The resulting mineralised scaffolds reached densities within the lower bounds of cancellous bone and demonstrated substantial mechanical reinforcement. DMA and PFM analyses confirmed increased compressive modulus and fibrillar stiffness associated with intrafibrillar mineral content. RFM is non-cytotoxic and supports cell growth; still its impact on osteogenic differentiation warrants further investigation for advanced bone modelling. Collectively, these findings establish RFM as a new platform for engineering rapid mineralised collagen scaffolds and expand current understanding of collagen–mineral interactions in synthetic bone design. Therefore, RFM scaffolds hold promise as synthetic bone substitutes and bone void fillers for critical-size defects, while providing valuable insights into the fundamental processes of collagen–mineral interactions.

## Data Availability

Data available in a publicly accessible repository via https://doi.org/10.25405/data.ncl.32420661, accessed on 15 July 2026.

## Supplementary Materials

The following supporting information can be downloaded at: https://www.mdpi.com/article/10.3390/jfb17070344/s1.
