# Supplementary material for: Bone-like Collagen Matrices Through Rapid Intrafibrillar Mineralisation
Source: J Funct Biomater. 2026 Jul 16;17(7):344. doi: 10.3390/jfb17070344 (PMC13413124; doi:10.3390/jfb17070344)
Supplement: Supplementary file 1 [file jfb-17-00344-s001.zip › jfb-4373215-supplementary.pdf]

Bone-Like Collagen Matrices through Rapid Intrafibrillar Mineralisation

(Supplementary data)

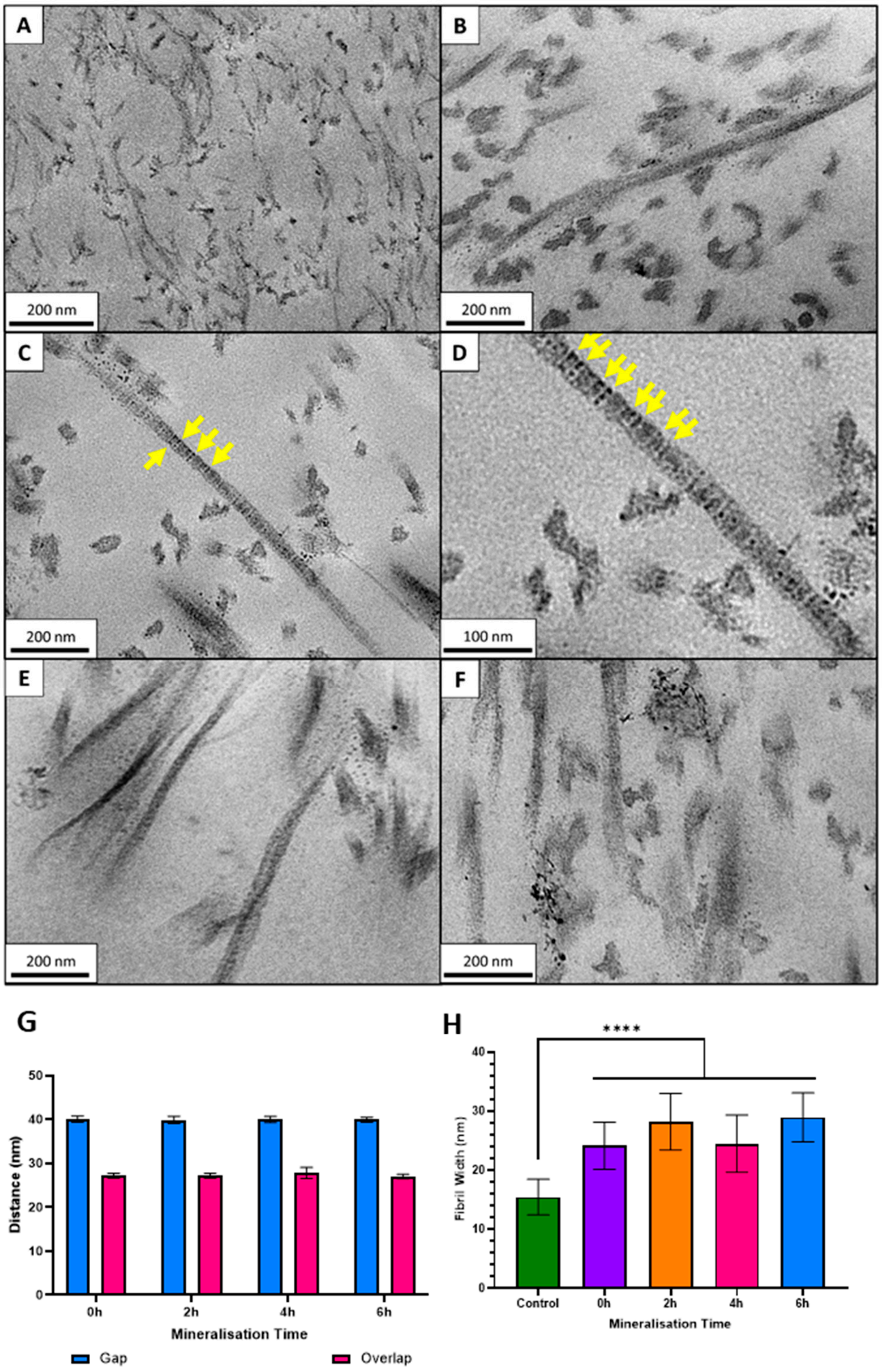

Figure S1. *TEM images of RFM samples and compared to a collagen control. (A) collagen control; (B) 0-hour RFM; (C, D) 2-hour RFM; (E) 4-hour RFM; (F) 6-hour RFM, (G) intrafibrillar mineral spacing of each sample group. No significant differences were found between the gap and overlap regions of each sample group. (H) Fibril width measurements from TEM images, bars represent the mean for each group, and the error bars are the individual standard deviations. A two-way ANOVA using the Bonferroni method was used.*

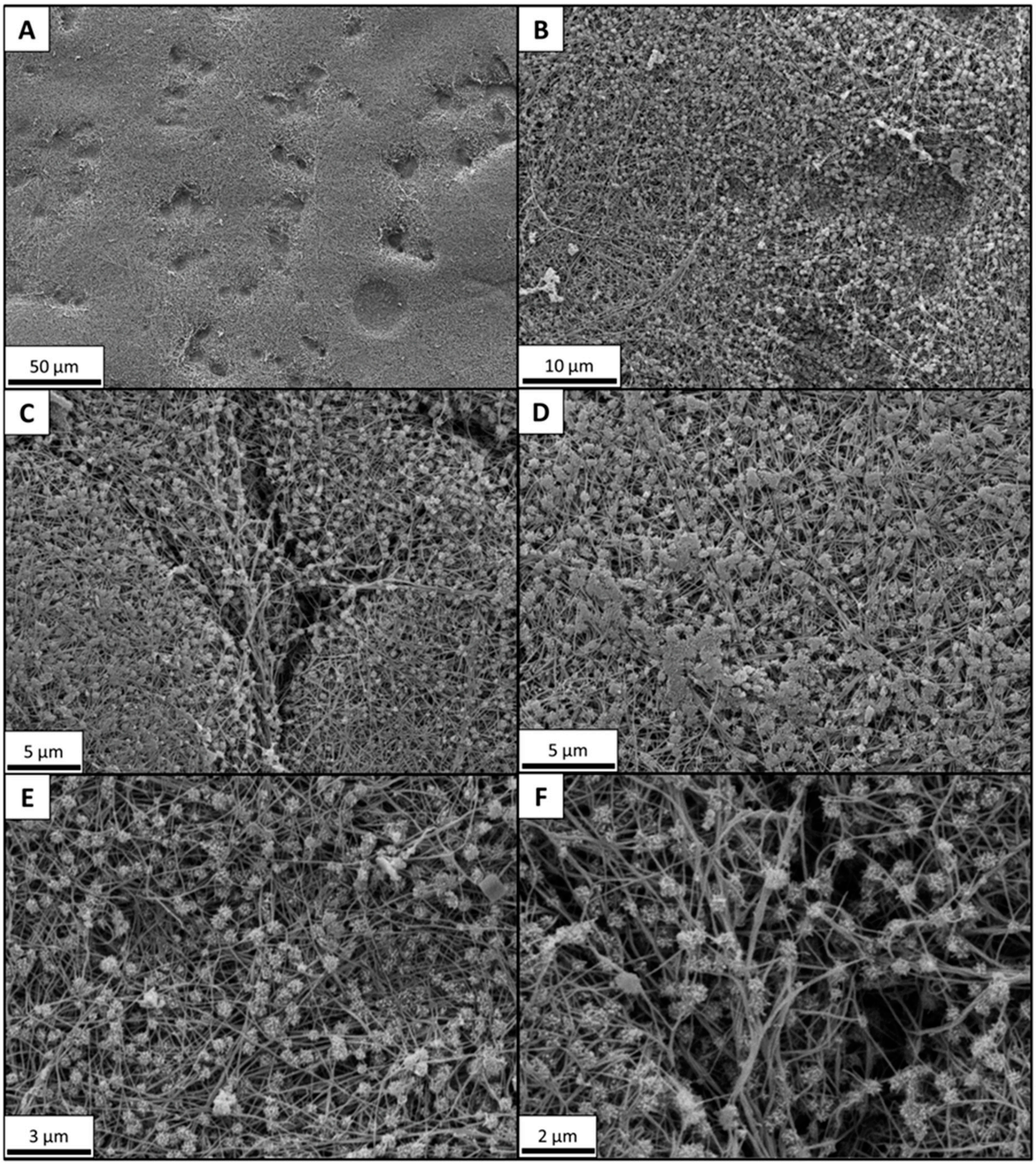

Figure S2. *SEM images of a 6-hour RFM sample. Multiple sites were imaged, and the magnification was gradually increased from A-F. Adjustments were made to brightness and contrast to achieve greater consistency between images*
